# The Protective Effect of Adiponectin-Transfected Endothelial Progenitor Cells on Cognitive Function in D-Galactose-Induced Aging Rats

**DOI:** 10.1155/2020/1273198

**Published:** 2020-03-23

**Authors:** Jing Huang, Botong Hou, Shuaimei Zhang, Meiyao Wang, Xuanzhen Lu, Qunfeng Wang, Yumin Liu

**Affiliations:** ^1^Department of Neurology, Zhongnan Hospital of Wuhan University, Wuhan 430071, China; ^2^Department of Neurology, The Second Affiliated Hospital of Nanchang University, Nanchang 330006, China

## Abstract

Aging is a multifactorial process involving the cumulative effects of inflammation, oxidative stress, and mitochondrial dynamics, which can produce complex structural and biochemical alterations to the nervous system and lead to dysfunction of microcirculation, blood-brain barrier (BBB), and other problems in the brain. Long-term injection of D-galactose (D-gal) can induce chronic inflammation and oxidative stress, accelerating aging. The model of accelerated aging with long-term administration of D-gal have been widely used in anti-aging studies, due to the increase of chronic inflammation and decline of cognition that similarity with natural aging in animals. However, despite extensive researches in the D-gal-induced aging rats, studies on their microvasculature remain limited. Endothelial progenitor cells (EPCs), which are precursors to endothelial cells (ECs), play a significant role in the repair and regeneration process of endogenous blood vessel, and adiponectin (APN), a protein derived from adipocyte, has many effects on protective vascular endothelium and anti-inflammatory. Recently, many studies have shown that APN can promote improvements in cognitive function. Under these circumstances, we investigated the neuroprotective effect of the APN-transfected EPC (APN-EPC) treatment on rats after administration with D-gal and explored the likely underlying mechanisms. Compared to model group for D-gal administration, better cognitive function and denser microvessels were significantly found in the APN-EPC treatment group, and indicated APN-EPC treatment in aging rats could improve the cognitive dysfunction and microvessel density. The level of proinflammatory cytokines IL-1*β*, IL-6, and TNF-*α*, activated astrocytes and apoptosis rate were significantly reduced in the APN-EPC group compared with the model group, showed that APN-EPCs alleviated the neuroinflammation in aging rats. In addition, the APN-EPC group inhibited the decrease of BBB-related proteins claudin-5, occludin, and Zo-1 in aging rats and attenuated BBB dysfunction significantly. These results of our study indicated that APN-EPC treatment in D-gal-induced aging rats have a positive effect on improving cognitive and BBB dysfunction, increasing angiogenesis, and reducing neuroinflammation and apoptosis rate. This research suggests that cell therapy via gene modification may provide a safe and effective approach for the treatment of age-related neurogenerative diseases.

## 1. Introduction

With the increase of aging countries and the proportion of aging population, there is a growing concern about aging. Aging is a normal physiological process and the leading cause of the cognitive decline, principally in learning, memory, sensibility, and perception functions. The mechanism may be related to chronic inflammation, oxidative stress, mitochondrial dysfunction, and other factors [1, 2]. Aging is accompanied with changes in the structure and function of microcirculation, resulting in vascular endothelial dysfunction and decrease of microvessels [2–4], thus, leading to age-related neurodegenerative diseases, such as vascular cognitive dysfunction and Alzheimer's disease (AD).

D-galactose (D-gal) is a reducing sugar found in many foods such as honey, yogurt, milk, and kiwi fruit. Excessive long-term D-gal intake can lead to the overproduction of advanced glycosylation end-products (AGEs) and reactive oxygen species (ROS) [5], which may contribute to chronic inflammation and oxidative stress [6]. Many studies have shown that chronic inflammation and oxidative stress would result in mitochondrial, neurological function damage, and even cognitive decline [7–12]. The model of accelerated aging with long-term injection of D-gal has been widely used in antiaging studies, due to the increase of chronic inflammation, decline of cognition, and biochemical indexes that are similar with natural aging in animals [5–13]. However, studies on the microvasculature in D-gal-induced aging rats remain limited.

Neurovascular unit (NVU), constitute by neurons, astrocytes, microglia, vascular endothelial cells, perivascular cells, basement membrane, and extracellular matrix, plays a very important role in maintaining the structure and function of the brain [14, 15]. Tight junctions (TJ) present between the endothelial cells of the capillaries that perfuse the brain parenchyma, assisted by astrocytic end-feet and pericytes, help maintain the stability of the blood-brain barrier (BBB) [15, 16]. As the production of ROS has been considered a key factor in the process of aging [17], it can induce neurovascular inflammation. Under the stimulation of chronic inflammation, the structure of NVU will be injured, which may result in activation of astrocytes, impairment of endothelial cell function, and cell apoptosis, which eventually leads to the decrease of microvascular density, increase of permeability of BBB, and impairment of nervous system function [16, 18–20].

EPCs, the precursor cells of endothelial cells derived from bone marrow, play a significant part in the process of endogenous blood vessel repair and regeneration [21]. EPCs can produce a variety of cytokines through paracrine to promote the proliferation and differentiation of endothelial cells (ECs) at the site of vascular injury and EPCs in circulation to repair injured vessels under the conditions of chronic inflammation, ischemia, and hypoxia [22–26]. In recent years, EPC transplantation has played an active role in the treatment of ischemic cerebrovascular diseases [25–27]. A previous study in our laboratory has also found that the treatment of middle artery occlusion (MCAO) rats with bone marrow-derived EPCs can play a good role in promoting angiogenesis, improving behavioral function, and reducing infarction area and apoptosis rate [27]. Nevertheless, the number of EPCs would decrease with aging [28], and the extraction of EPCs is difficult as well. When transplanted alone, just a few EPCs survived, which weaken the process [29].

Adiponectin (APN), a protein synthesized by adipocyte, plays an important role in the regulation of glucose and lipid metabolism. APN also has been proposed to have essential functions like vascular endothelial protection, anti-inflammatory, antiatherosclerosis, and vasodilatory properties which may influence the central nervous system disorders. The content of APN was positively correlated with the level of EPCs [30, 31], and APN can also be involved in regulating the function and promoting the role of EPCs [32–36]. In recent years, a growing number of studies have shown that APN can promote improvements in cognitive function [37, 38].

Therefore, we transfected APN gene into EPCs by lentivirus through genetic engineering technology to make EPCs overexpress APN, then the APN-EPCs are supposed to play a better role in endothelial repair and anti-inflammation to make a positive effect on cognitive function in D-gal-induced aging rats.

## 2. Materials and Methods

### 2.1. Preparation of EPCs

4-week-old male Sprague-Dawley (SD) rats provided by the Centers for Disease Control of Hubei Province were anesthetized with 3% pentobarbital sodium at 30 mg/kg and then killed, isolated the tibia and femur of the rats aseptically, and rinsed their bone marrow cavities with PBS buffer repeatedly to collect fresh bone marrow. Mononuclear cells were isolated by density gradient centrifugation using the Ficoll-Paque solution (*d* = 1.077, Sigma, USA). Then, they were inoculated in a T25 culture flask with endothelial cells basal medium-2 (EBM-2, Lonza, Switzerland) and placed in a cell incubator with 5% CO_2_ atmosphere at 37°C. Changing the media every 72 hours and the growth state of the cells were observed every day. In our previous experiments [27], we identified these cells by the property of uptaking acetylated LDL (ac-LDL) and Ulex europaeus agglutinin-1 (UEA-1) by EPCs, and cells cultured by this method have been identified as EPCs.

### 2.2. Gene Transfection

EPCs were collected after a 9-day culture and transfected with lentiviral vectors encoding the human APN gene (LV-APN/EGFP, Shanghai Genechem Company, China) at a transfection multiple of 100. The vector of this gene is a relaxed shuttle plasmid, which can be amplified without restriction, and the sequence of its vector elements is Ubi-MCS-3FLAG-SV40-EGFP-IRES-puromycin. After 12 hours, the cells were washed with PBS buffer and cultured with fresh media. 72 h after transfection, the cells were placed under an inverted fluorescence microscope to observe the expression of enhanced green fluorescent protein (EGFP) and evaluate the results of transfection, and the transfection rate was calculated according to the following formula: transfection rate = positive cells/total cells per field × 100%. The expression of APN from APN-EPCs and EPCs were detected by Western blot analysis [27].

### 2.3. Animals and Drug Administration

48 male SD rats, weighing 200-220 g, provided by Beijing Vital River Laboratory Animal Technology Company were used in our experiments. The animal study proposal was approved by the Institutional Animal Care and Use Committee (IACUC) of Wuhan University (Hubei, China) with the permit number IACUC 2019060. All the operations and handling of experimental animals are complied with the requirements of animal ethics strictly.

The rats were randomly separated into four groups: (1) control group (*n* = 12), (2) model group (*n* = 12), (3) EPC treatment group (*n* = 12), and (4) APN-EPC treatment group (*n* = 12). Rats in the model group and the treatment group were given intraperitoneal injection with 100 mg/kg D-gal daily for 6 weeks, while the control group was given intraperitoneal injection of normal saline at the same dose every day for 6 weeks.

### 2.4. Cell Transplantation

The EPCs cultured for 14 days and the APN-EPCs cultured for 5 days were resuspended with PBS buffer and collected, respectively. After 6 weeks of intraperitoneal injection, 0.5 ml EPC and APN-EPC suspension containing 2 × 10^6^ cells were injected into the EPC treatment group and APN-EPC treatment group, respectively, through the tail vein, while 0.5 ml PBS buffer was injected into the control group and model group.

### 2.5. Morris Water Maze Test

All the rats were given spatial memory assessment by using Morris water maze (MWM), which mainly includes a 1.5 m diameter pool filled with opaque water and a 10 cm round platform that placed 1 cm below the surface of the water at the center of one quadrant. The whole experiment lasted for 6 days. The first 5 days were the place navigation test, which was used to test the spatial learning and memory functions of rats. During the first 5 days, each rat was gently released into the water at four different locations on opposite sides of the platform for four swimming trials per day. Rats that failed to find the platform within 60 s were manually guided to the platform and allowed to stay for 15 s. The probe trial proceeded on the 6th day, which was tested for spatial memory. On the 6th day, the platform was removed, the contralateral side of the platform was selected as the entry point, and the rats were free to swim in the tank for 60 s. The swimming trace, latency to the platform, the time spent in each quadrant, and the platform crossing times were recorded by a computerized video imaging analysis system (AVTAS Animal Video Tracking Analysis System, Wuhan YiHong Sci. & Tech. Co., Ltd).

### 2.6. Tissue Preparation

After behavior testing, the rats were anesthetized with 3% pentobarbital sodium. Six rats were randomly selected from each group and transcardially perfused with 4% paraformaldehyde solution. The brains were postfixed overnight in 4% paraformaldehyde and embedded in paraffin after full dehydration. Then the brains were cut at 4 *μ*m thick coronal sections from the anterior fontanelle -3.84 mm to -5.04 mm, and the sections were dewaxed before use. Brains of the remaining rats were taken after decollation, then, the bilateral hippocampal tissues of these brains were isolated on the ice rapidly and transferred to a refrigerator at -80°C for storage immediately.

### 2.7. Western Blotting

The rat hippocampal tissues were homogenized in lysis buffer with phenylmethanesulfonyl fluoride (PMSF) and phosphatase inhibitor single-use cocktail (Beyotime, Shanghai, China), followed by 30 min on ice. The lysates were centrifuged at 12,000 rpm for 5 min at 4°C, and the supernatant solutions were collected. The total protein concentration of each sample was determined using a BCA protein assay kit (Beyotime, Shanghai, China). Then, the lysates were separated by the sodium dodecyl sulfate-polyacrylamide gel electrophoresis (SDS-PAGE) and transferred to nitrocellulose membranes. The membranes were incubated in 5% nonfat milk in PBS with 0.1% Tween-20 at room temperature (RT) for 1 h. Probing with primary antibodies to claudin-5 (Biorbyt, Wuhan, China), IL-6, IL-1*β* (Bioss, Beijing, China), glyceraldehyde 3-phosphate dehydrogenase (GAPDH), occludin, Zo-1, and TNF-*α* (Abcam, UK), followed by incubation with horseradish peroxidase-conjugated goat anti-rabbit IgG and detection using a chemiluminescence substrate. The AlphaEaseFC software was used for analysis, and the optical density values of the target proteins detected in each experimental group were normalized to the optical density values of the GAPDH control groups.

### 2.8. Immunohistochemical Analysis

The brain sections were treated with 0.1% Triton X-100 for 10 min, then, washed with 0.1 M PBS. Endogenous peroxidase activity in the brain sections were blocked with 3% hydrogen peroxide, then blocking the nonspecific binding sites with 3% normal goat serum at RT for 30 min. After blocking, the sections were incubated overnight in rabbit anti-caspase-3 and anti-glial fibrillary acidic protein (GFAP) antibodies (Proteintech, Wuhan, China), then incubation with horseradish peroxidase-conjugated goat anti-rabbit IgG. The sections were washed with PBS and incubated in 3,3 0-Diaminobenzidine tetra hydrochloride (DAB), rinsing the sections with running water to stop color development, and brown or tan staining on the cell membrane or in the cytoplasm represented positive staining. The density of positive staining was the expression levels of caspase-3 and GFAP proteins, and the results were measured by the ImageJ software.

### 2.9. Immunofluorescence Analysis

The brain sections were washed three times with 0.1 M PBS for 3 min after treating with 0.1% Triton X-100 for 10 min. Blocking the nonspecific binding sites with 3% normal goat serum at RT for 30 min, then the sections were incubated with the primary antibody (CD31, 1 : 100, Abcam) overnight at 4°C washing with PBS buffer, the sections were incubated with fluorescein isothiocyanate- (FITC-) labeled goat anti-rabbit IgG secondary antibodies (1 : 100, Boster, China) for 1 h. The ImageJ software was used to analyze immunohistological quantitative.

### 2.10. TUNEL Staining

The brain sections were washed three times for 10 min in proteinase K solution (20 *μ*g/ml) which was diluted with PBS buffer. Apoptotic cells in the sections were detected by a transferase-mediated dUTP-biotin nick end labeling (TUNEL) apoptosis detection kit (Yeasen, Shanghai, China), then incubating with DAPI in a dark environment for 5 min. The number of apoptotic cells was observed and counted under the fluorescence microscope, and the apoptosis rate was calculated according to the following formula: apoptosis rate = positive cells/total cells per field × 100%.

### 2.11. Statistical Analysis

The GraphPad Prism 8.0 software was used for statistical analysis. The data was presented as the mean ± standard error of mean (SEM). Two-way analysis of variance (ANOVA) was used to analyze the escape latency in the MWM training task, and the other data was analyzed by a one-way ANOVA. The value with *p* < 0.05 was considered to be statistically significant.

## 3. Results

### 3.1. EPC Morphology and Transfection Result

**Figure 1:**
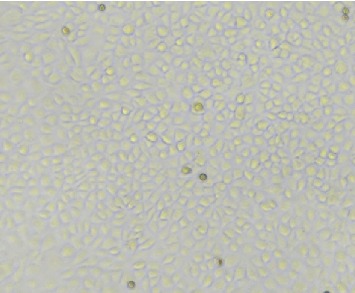
The EPCs were cultured for 14 days were arranged like pebbles.

**Figure 2:**
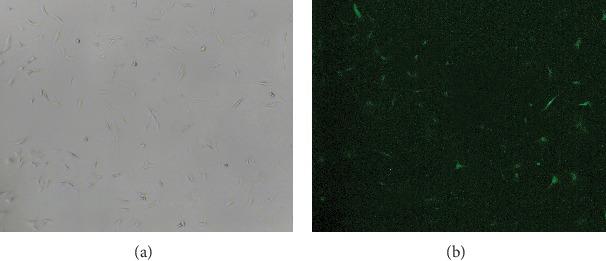
The transfection result. (a) The EPCs observed under a microscope in natural light (x100). (b) The positive cells with green fluorescent protein (GFP) were observed by fluorescence in the same field and at the same magnification.

We observed with the microscope that the newly extracted mononuclear cells were small, round, and suspended in the medium. The cells were arranged like pebbles after 14 days of culture (Figure 1). After 72 hours of gene transfection, the cells were placed under a fluorescence microscope (×100), and a large number of EPCs with green fluorescence were observed, which manifested that the transfection was successful (Figure 2). The ImageJ software was used for cell counting and analysis, and the transfection rate was 71.3 ± 8.8%.

### 3.2. The APN-EPCs Prevent D-Gal-Induced Cognitive Impairment

**Figure 3:**
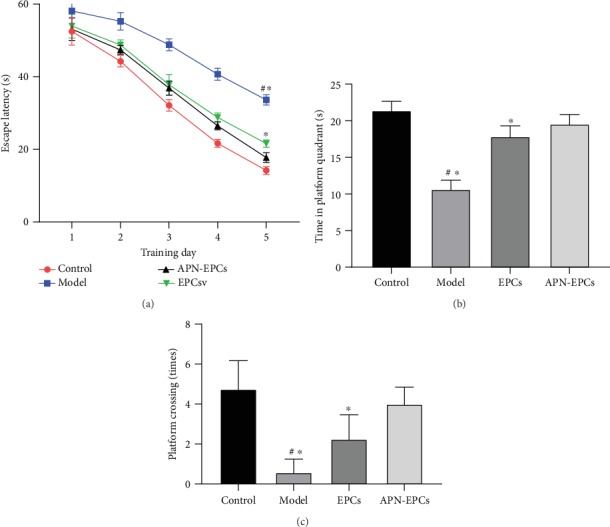
Influence of APN-EPC treatment on MWM test of aging rats. (a) The place navigation test. (b) The probe trial. (c) Crossing times. ^∗^*p* < 0.05 the model group or the EPC group compared with the APN-EPC group, ^#^*p* < 0.05 the model group compare with the control group.

The results of the study showed that the APN-EPCs significantly ameliorated the memory deficits of D-gal-induced aging rats (Figure 3). In the place navigation test, the escape latency of the model group was significantly increased compared with the control group (*p* < 0.05), the treatment groups were significantly shorter than that of the model group (*p* < 0.05), and there was a significant difference among the four groups (F (12, 220) = 19.42, *p* < 0.01). Compared with the EPC treatment group, the escape latency of the APN-EPC treatment group was significantly shortened, and the difference was statistically significant on day 4 and day 5 (*p* < 0.05, Figure 3(a)). In the probe trial, the time of the rats in the model group spent in the platform quadrant was significantly shorter than that in the control group (*p* < 0.05), and the duration of the rats in the APN-EPC treatment group in the platform quadrant was significantly longer than that in the EPC treatment group and the model group (*p* < 0.05, Figure 3(b)). An increase time of platform crossing was observed with the APN-EPC and EPC treatment group compared with the D-gal treated group (Figure 3(c), *p* < 0.05). These results indicated that APN-EPC treatment prevented D-gal-induced cognitive impairment.

### 3.3. The APN-EPCs Attenuate D-Gal-Induced Neuroinflammation

**Figure 4:**
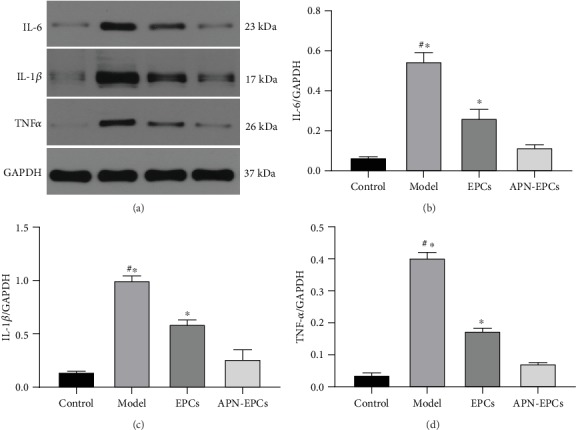
Influence of APN-EPC treatment on hippocampal inflammatory cytokines of aging rats. (a) Western blotting results of inflammatory cytokines. (b) Quantitative result of IL-6. (c) Quantitative result of IL-1*β*. (d) Quantitative result of TNF-*α*.^∗^*p* < 0.05 the model group or the EPCs group compared with the APN-EPC group, ^#^*p* < 0.05 the model group compare with the control group.

**Figure 5:**
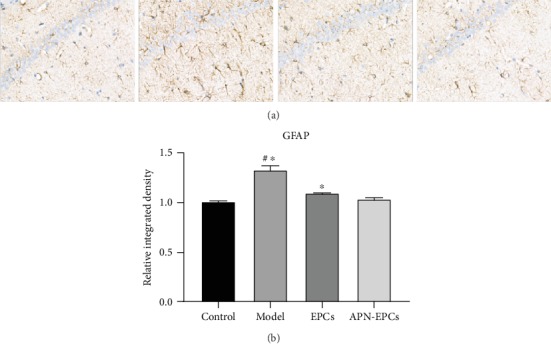
Influence of APN-EPC treatment on hippocampal astrocytes. (a). Immunohistochemistry result of GFAP in the hippocampus (x400). (b). Quantitative result of GFAP. ^∗^*p* < 0.05 the model group or the EPC group compared with the APN-EPC group, ^#^*p* < 0.05 the model group compare with the control group.

The Western blotting results showed that significantly increased protein levels of IL-1*β*, IL-6, and TNF-*α* in the aging rat hippocampus when compared with the control group (*p* < 0.05, Figure 4). In the immunohistochemical analysis, the expression of GFAP, which is a specific marker for activated astrocytes, increased significantly in the model group when compared with the control group (*p* < 0.05, Figure 5). The APN-EPC group prevent the increase of inflammatory cytokines and GFAP in the model group, and the effect was better than that of EPC group (*p* < 0.05). These results indicated that APN-EPC treatment prevented D-gal-induced neuroinflammation.

### 3.4. The APN-EPCs Attenuate D-Gal-Induced BBB Dysfunction

**Figure 6:**
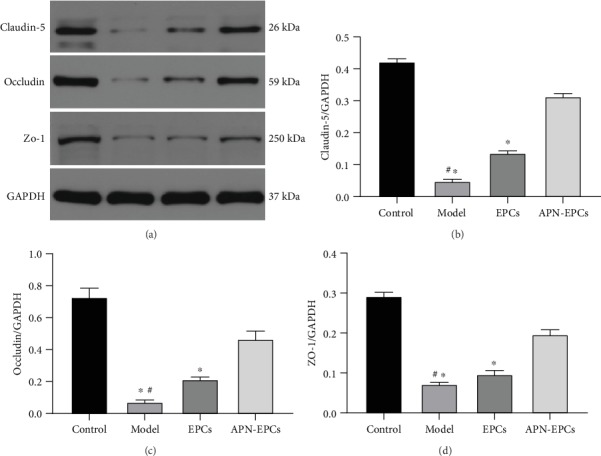
Influence of APN-EPC treatment on BBB function. (a). Western blotting results of the BBB-related proteins. (b). Quantitative result of claudin-5. (c). Quantitative result of occludin. (d). Quantitative result of Zo-1.^∗^*p* < 0.05 the model group or the EPC group compared with the APN-EPC group, ^#^*p* < 0.05 the model group compare with the control group.

Claudin-5, occludin, and Zo-1 are proteins related to the tight junctions (TJ) in the BBB; they play an important role in the size-selective relaxation of the BBB together. We observed the Western blotting results that the expression of claudin-5, occludin, and Zo-1 in the aging rat hippocampus were significantly decreased when compared with the control group (*p* < 0.05). The APN-EPC group increases the expression of the proteins in the model group, and the effect was better than that of EPC group (*p* < 0.05, Figure 6).

### 3.5. The APN-EPCs Can Improve the Microvessel Density

**Figure 7:**
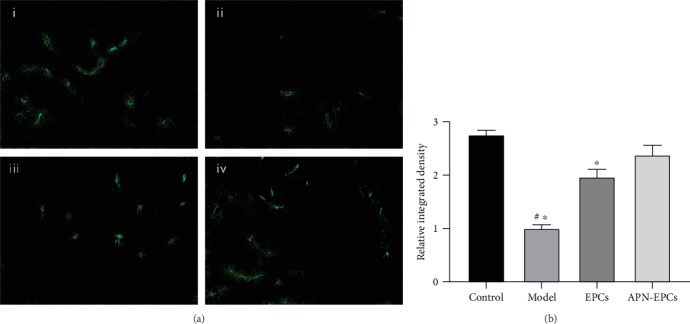
Influence of APN-EPC treatment on microvessel density. (a). CD31 immunofluorescence staining in the hippocampus (x400); (i) control group, (ii) model group, (iii) EPC group, and (iv) APN-EPC group. (b). Quantitative result of microvessel density.^∗^*p* < 0.05 the model group or the EPC group compared with the APN-EPC group, ^#^*p* < 0.05 the model group compare with the control group.

We observed through immunofluorescence microscopy that the microvessel density in the hippocampus of the aging rats treated with APN-EPCs was higher than that in the EPC treatment group and the model group (*p* < 0.05, Figure 7).

### 3.6. The APN-EPCs Attenuate the D-Gal-Induced Cell Apoptosis

**Figure 8:**
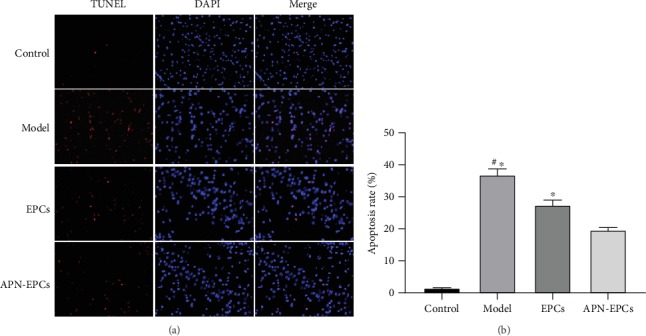
Influence of APN-EPC treatment on apoptosis rate. (a). TUNEL/DAPI immunofluorescence staining (x400). (b). Cell apoptosis rate.^∗^*p* < 0.05 the model group or the EPC group compared with the APN-EPC group, ^#^*p* < 0.05 the model group compare with the control group.

The results in the TUNEL staining showed that apoptosis rate in the model group was significantly increased compared with the control group (*p* < 0.05), while that of the APN-EPC treatment group was decreased when compared with the EPC treatment group and model group (*p* < 0.05, Figure 8).

## 4. Discussion

In this research, we induced subacute aging rats by excessive and long-term intraperitoneal injection of D-gal, which resulted in cognitive dysfunction, decreased brain microvascular density, increased apoptosis rate and release of proinflammatory cytokines, activation of astrocytes, and decreased BBB-related proteins. After the injection of APN-EPCs through the tail vein, we observed that the cognitive function was improved, the density of brain microvascular was increased, the function of the BBB was improved, the apoptosis rate and the level of proinflammatory cytokines was decreased in the aging rats, and the effect was better than that of EPCs.

In D-gal-induced aging rat model, the excessive and long-term injection of D-gal can lead to the overproduction of AGEs and ROS, which may result strong oxidative stress and chronic inflammation that accelerates aging in rats [9–13]. Chronic, aseptic, and low-degree inflammation was considered a sign of the aging process [2]. So, we focused on the inflammation cytokines in the aging rats. Interleukin-1 (IL-1) family of cytokines are key mediators of the inflammatory response, and tumor necrosis factor-alpha (TNF-*α*) is a proinflammatory cytokine that is generally produced by microglia and astrocytes in the brain [6]. In our study, we have shown the increase of the proinflammatory cytokines IL-1*β*, IL-6, and TNF-*α*, and the activation of astrocytes in the D-gal-induced aging rats. The results are just consistent with the previous study [6, 8, 9].

Moreover, the neuroinflammatory responses induced by ROS can lead to structural injured of NUV, such as activation of astrocytes, impairment of endothelial cells function, and apoptosis of cells, which has been thought to result in BBB dysfunction [39, 40]. Endothelial TJ density proteins, such as claudin-5, together with integral membrane proteins and cytoplasmic proteins, such as occludin and Zo-1, are involved in maintaining the permeability of BBB. Matrix metalloproteinase (MMP) is overproduced due to oxidative stress and chronic inflammation induced by age-related vascular changes, it can facilitate the degradation of TJ proteins and the basement membrane [15], thus, leading to increased permeability of the BBB. Impaired BBB function has been associated with neurodegeneration and cognitive decline [18, 19]. Our study just indicated the cognitive dysfunction and the BBB-related proteins claudin-5, occludin, and Zo-1 increased in the subacute aging model.

EPCs, derived from bone marrow, play a significant part in the process of endogenous blood vessel repair and regeneration. However, the number of EPCs decreases accompany with aging [28], and difficulties still exist to obtain the EPCs. As is known, APN is a kind of adipocytokine, which has the functions of protecting vascular endothelium, anti-inflammation, and antiatherosclerosis [41, 42]. It can also be involved in regulating the function and promoting the role of EPCs. In the study of Lavoie et al. [33], APN could regulate the ability of EPCs to repair blood vessels by reducing the apoptosis of EPCs and enhancing their functions. Wang et al. [34] and Dong et al. [35] found that APN can enhance the function of EPCs through mTOR-STAT3, AMPK/eNOS, and other signaling pathways. In this study, we transfected APN gene into EPCs through genetic engineering technology and found that APN-EPCs have effects on reducing proinflammation markers, improving cognitive function, microvascular density, and structural damage of aging rats, and the effect was significantly better than EPCs transplanted alone.

Our study demonstrated that the treatment of APN-EPCs inhibited proinflammatory gene expression and prevented neuroinflammation in aging rat, it may relate to the anti-inflammatory effect of APN. Under the anti-inflammatory and vascular endothelial protective effects of APN, the content of BBB-related proteins gradually recovered and the injury of endothelial cells decreased. APN-EPCs also increased microvascular density in aging rats, it may be due to the endogenous blood vessel repair and regeneration of EPCs. With the recovery of microvascular density and the increase of BBB-related proteins, the BBB integrity was restored, which eventually lead to the improvement of cognitive function. The likely mechanism is that under the environment of chronic inflammation and oxidative stress, APN-EPCs in aging rats acted on the impaired areas of vascular endothelium through a variety of paracrine cytokines to participate in vascular repair and regeneration. Moreover, it plays an anti-inflammatory and antiapoptotic role in improving brain microcirculation, BBB function, and nervous structure damage.

## 5. Conclusion

Our data demonstrated that the APN-EPC treatment had a positive effect on nervous structure damage and cognitive dysfunction by repairing injured blood vessels and reducing neuroinflammation. The results suggested that gene-modification cell therapy may provide a safe and effective approach for the treatment of age-related neurogenerative diseases.

## Data Availability

The data used to support the findings of this study are included within the article.
